# Is it early enough? The authentic meaning of the pediatric palliative approach between early and late referral in pediatric oncology: a case study

**DOI:** 10.3389/fonc.2024.1397983

**Published:** 2024-07-25

**Authors:** Anna Santini, Irene Avagnina, Maria C. Affinita, Anna Zanin, Franca Benini

**Affiliations:** ^1^ Pediatric Pain and Palliative Care Service, Department of Women’s and Children’s Health, School of Medicine and Surgery, University of Padua, Padua, Italy; ^2^ Division of Pediatric Hematology, Oncology and Stem Cell Transplant, Department of Women’s and Children’s Health, School of Medicine and Surgery, University of Padua, Padua, Italy

**Keywords:** pediatric palliative care (PPC), early referral to palliative care, integration of care, pediatric oncology and hematology, team communication

## Abstract

The literature widely supports the benefits of early integration of palliative care into pediatric oncological care; however, many barriers to its successful integration remain. Integrating palliative care as early as possible in the oncology pathway is critical, but other criteria are relevant to positive results. This paper aims to contribute to the early/late referral dualism in pediatric palliative care (PPC) and highlight the importance of a collaborative approach between oncologists and palliative care teams. This study investigates the impact of early versus late referral to PPC, intersecting it with the synergy work between services and the related outcomes. The four pediatric cancer cases were selected based on clinical (e.g., disease duration, multiple treatments, and pain management), management (e.g., involvement of multiple services and multiple home–hospital transitions), and relevance of multidisciplinary team (e.g., difficult clinical decisions and ethical discussions) criteria. A mixed-methods approach was employed, combining qualitative case analysis using clinical diaries, literature review, and practice guidelines development. Critical clinical information, time course, clinician–family communication, and patient involvement were analyzed. The outcomes show how simultaneous care creates continuous discussion and dialogue between professionals. The results indicate the importance of better communication and care coordination to improve patient and family satisfaction, highlighting the uniqueness of the pediatric field and the relationship with children and families. Through the discussion of clinical cases and a literature review, we provide practical guidance for clinicians working in oncology and PPC. These findings underscore the crucial need for a multidisciplinary approach in pediatric oncology, advocating policy changes to support early PPC integration and translate it into complementarity best operating practices. In conclusion, besides assessing the timeliness of referral to the PPC service, the synergy, harmony, and choral work of the professionals involved are equally valuable for a quality-of-life-oriented care plan.

## Highlights

- Training on criteria and protocols to facilitate case reporting by oncologists to the PPC team.
- Using validated and shared tools and scales to assess the complexity of patient and family needs.
- Giving voice to all professionals (e.g., nurses, family physicians, and psychologists). Needs can be collected from everyone, and any healthcare figure can propose referrals to the PPC service.
- Always maintaining collaboration between services and implementing synergistic work between oncology and palliative care.

## Introduction

In the late 1990s, the World Health Organization (WHO) and the American Academy of Pediatrics proposed the early integration of palliative care in pediatric patients with cancer at diagnosis, regardless of prognosis (1). Over the past 20 years, many studies (2–5) have reported the benefits of this approach in terms of improved quality of life, support of more family-centered communication, enhanced assessment and management of physical symptoms and distress, and identification of psychosocial concerns and spiritual considerations of the patient, parents, and siblings.

Although the literature supports the positive effects of early integration of palliative care principles into pediatric oncological care, many barriers hinder the success of this inclusion. The systematic review by Cheng et al. showed that 54.5% of pediatric patients with cancer still do not receive pediatric palliative care (PPC) support before death (2). The EUROCARE-6 study recently reported long-term survival for childhood cancers in Europe (6), pointing out that still more than 1,600 patients died of cancer in Europe in the last year. Similar data were recorded in the US, where approximately 10% of children and adolescents (age 0–19 years) with cancer were expected to die in 2023 (7).

Despite the growing understanding of palliative care principles and the increasing interest of the scientific and public community over the past 20 years, many barriers continue to limit access to and expansion of PPC and hospice services worldwide. Personal and institutional barriers have been described in the literature: misconceptions about palliative care as an under-recognized specialty; lack of qualified palliative care providers, resulting in high demand that leads to overburdened PPC teams; inadequate education and training in palliative care; financial barriers; lack of structured home-based services; PPC programs only on weekdays, during office hours, with limited in-home and outpatient access to palliative care; and resistance from family members. Because of these reasons, the early integration of palliative care into the pediatric oncology service is currently the subject of much debate.

Early referral to PPC is regarded as a standard goal; however, it cannot be the only one. Physicians also stress the need for multidisciplinary collaboration among pediatric professionals, as PPC is still considered end-of-life (EoL) care with no possibility of simultaneous care integration (8).

At present, the gap in the literature focuses mainly on the timing of PPC introduction. Effective integration of PPC requires robust multidisciplinary collaboration, as isolated PPC efforts often fail to meet the comprehensive needs of pediatric oncology patients. Our scientific work intends to move beyond the concept of early referral and integrate it with a broader vision of maintaining linkages and working synergistically among services. Standardizing practices can be problematic because PPC patients often have high heterogeneity. PPC case reports can help show this heterogeneity and the complexity of clinical cases.

Our study aims to highlight how early referral alone does not guarantee simultaneous care, as it is not sufficient to achieve synergy and sharing of care goals among different clinical teams. In our experience, we have a solid and ongoing collaboration and exchange with the pediatric onco-hematology service. Through our collective efforts, we have tackled critical care issues and made a real difference in the lives of our patients. This article showcases some of the cases we discussed, highlighting teamwork’s importance in achieving positive outcomes. By working collaboratively, we can continue to improve and grow as a team, providing the best possible care for children in need.

This study analyzes early and late referrals and the achievement of integration or discontinuity of care through four clinical cases. The researchers recruited all cases from the Department of Women’s and Children’s Health—University of Padua.

The four pediatric cancer cases were selected based on clinical, management, and relevance of multidisciplinary team criteria. Clinical considerations included tumor type (solid tumors), multiple treatments (presence of surgery and chemotherapy or radiotherapy), disease duration (<48 months), active treatment duration (<48 months), need for multiple treatments, and initial involvement of PPC as pain control. Management criteria included multiple service involvement, transitions between home and hospital settings, and consideration of multiple levels of care. Multidisciplinary team relevance criteria included case discussions regarding the clinical appropriateness of interventions, bioethics, the child’s best interest, and the family’s quality of life. A mixed-methods approach was employed, combining qualitative case analysis using clinical diaries, literature review, and practice guidelines development.

## Case 1—Ida (late referral and discontinuity of care)

Ida was a 14-year-old girl with malignant mediastinal schwannoma while already suffering from neurofibromatosis type 1.

She was 13 years old at the onset of the disease, lasting a total of 12 months from diagnosis to death. At diagnosis, Ida underwent intensive chemotherapy (CT) for 7 months; 9 months after the diagnosis, she underwent excisional surgery.

As the PPC team, we met Ida 1 month after the excisional surgery in the context of an antalgic consultation for neuropathic pain in the left upper limb.

In the following weeks, Ida presented with progressive clinical deterioration with the appearance of hemothorax, hemoptysis, and chest pain. Ida was hospitalized three times in 5 weeks in three different wards; she underwent bronchial lavage, CT angiography, and further diagnostic investigations until a PET-RMN was performed 10 days before her death. PET-RMN showed disease dissemination at the pleural and bone levels. The onco-hematologist informed the mother of the progression of the disease and its incurability. Ida began receiving continuous infusions of morphine 1 week before death (without adequate pain control). The oncologist involved the PPC team in the conversation on terminality with the mother; given worsening symptoms and poor pain control, the patient was transferred to a hospice for EoL care. Once assigned to our unit, pain control was finally achieved. Despite the shortness of her pediatric hospice stay, she reported that she had adequate pain control and a substantial decrease in dyspnea symptoms for the first time in the last 6 months; moreover, she was able to walk again. During the hospice stay, both parents and many members of the family could stay with her; this was a change from the situation experienced during the previous 6 months of hospitalization, where visits from relatives and friends had been suspended at the family’s request. Psychological support was proposed and accepted by the family.

The PPC center was never officially activated, and as palliative care providers, we only participated in one joint communication, which led to the transfer from the oncology unit to our hospice. Ida died 2 days after being transferred to hospice with reasonable pain control. See **Table 1** for a summary of the main points of this case.

**Table 1:** Summary of case highlights.

Reason for referral: a terminal disease with difficulty in controlling symptoms
The PPC team fully introduced themselves to the parents and as a pain service to the patient
Late referral
The patient was transferred to pediatric hospice during EoL care
+ Well-controlled symptoms during EoL care - Lack of the patient’s involvement in redirecting care goals and communication; delayed pain management

## Case 2—Antony (late referral and integration of care)

Antony was a 3-year and 10-month-old child with embryonal rhabdomyosarcoma localized at the temporal–sphenoidal level and with meningeal dissemination at diagnosis. At the onset, Antony had undergone excisional surgery, followed by radiotherapy (RT) and CT. During treatment, approximately 12 months after onset, Antony presented with altered consciousness, dysarthria, and gaze deviation; magnetic resonance imaging (MRI) findings showed a disease recurrence in urgent care.

At that time, the suburban hospital near the patient’s home presented the case to the PPC service after having told the family that the disease was incurable. The oncology team asked the PPC team to participate in an online meeting with the family, the local hospital pediatric oncologist team, and a highly specialized pediatric oncology team from the central hospital. On this occasion, the family was informed that the disease had progressed rapidly, even with the risk of imminent death. Online communication was unstable, and the conversation occurred in the presence of the child, who did not want to detach himself from his mother. The oncology team proposed high-dose CT to extend life expectancy but shared the possible side effects. The palliative care service introduced itself without the opportunity to discuss the treatment plan.

At the end of this conversation, the parents chose to try palliative CT and asked to stop it if the side effects were intolerable. The family requested to take a trip to the mountains for a few days before hospitalization, and PPC service managed and supported the family with everything they needed to fulfill this wish.

Antony lived for the next 2 months. He underwent a course of CT for 2 weeks, after which, in the absence of clinical improvement, it was discontinued. During these treatments, Antony remained in the oncology ward, in a double room with other pediatric patients, and with the continuous care of his mother. The PPC psychologist reported his mother’s great tiredness and difficulty with any activity, as Antony was whiny, irritable, and difficult to distract. Subsequently, the child returned home, and treatment was interrupted due to side effects.

Two weeks before death, the PPC team held a home consultation on shared care planning with the family, and it was agreed that no resuscitative maneuvers would be performed in case of emergency/urgency. The family’s quality of life improved by allowing them to stay at home with no further access to the hospital. Following the last unsuccessful palliative CT treatment, the family decided to prioritize Antony’s wishes. The parents wanted to be able to manage the worsening of symptoms and the accompanying death with the territorial support services coordinated by the PPC service. The parents lived close to their grandparents and other relatives from whom they received practical and emotional support. Psychological support was always available, but the parents did not use it.

The care process was carried out with the coordination of the PPC team exclusively through territorial services, respecting the family’s wishes. Palliative sedation was performed at home with complete symptom control until death. See **Table 2** for a summary of the main points of this case.

**Table 2:** Summary of case highlights.

Reason for referral: disease progression and terminality
The PPC team fully introduced themselves
Late referral
Clinical services maintained shared care direction
+ Coordination between services enabled the family’s wish for the patient to die at home - PPC service was omitted in the discussion of palliative treatment choices

## Case 3—Gabriella (early referral and integration of care)

Gabriella was a 6-year and 6-month-old girl with diffuse midline glioma.

The oncological diagnosis occurred at the age of 5 years and 8 months, and the disease lasted a total of 10 months.

At the time of diagnosis, Gabriella presented with VII cranial nerve paralysis and hemi-inattention, with acute deafness. One month after diagnosis, Gabriella underwent neurosurgical partial excision surgery, resulting in altered consciousness, generalized hyposthenia, and dysphagia, i.e., feeding through a nasogastric tube and absence of verbal communication.

Gabriella was referred early to the PPC network 2 months post-onset after a long-term post-intervention stay in the pediatric intensive care unit and admission to pediatric onco-hematology, where CT was started every 15 days.

The oncology team presented the case to the PPC team during the child’s admission to their unit. The parents were invited to a dedicated interview to explain the support of the PPC team and to communicate a transition to their hospital ward for parental empowerment about Gabriella’s care needs before returning home. The parents accepted the PPC service but with the explicit request to maintain hope that the neurosurgical operation would give Gabriella time and perhaps even a possible cure. The family’s quality of life relied on intensive rehabilitation, and Gabriella’s improvements were a source of joy and hope.

Gabriella was discharged and returned home after 2 months in the hospital.

In the following months, Gabriella progressively recovered her interpersonal and motor skills. She resumed eating independently, walking with support, and speaking. It was possible to envision the activation of a home rehabilitation service. Gabriella’s wishes were to return to school and to be able to go to an amusement park during the Christmas season with some friends.

The PPC service operated in all settings where Gabriella was involved (e.g., home, school, friends’ house, and amusement park). An experienced psychologist provided psychological support from the PPC service to both the mother and Gabriella. Since Gabriella liked drawing, this activity was used as a psychological strategy to manage her emotions. The relatives from the maternal branch were of considerable practical and emotional support; therefore, the PPC service involved them in informational and psychological support interviews. Gabriella’s parents were separated; the mother’s new partner, who was very close to Gabriella, was present. The PPC team effectively coordinated all the care settings: the oncology day hospital, rehabilitation center, hospice, and territorial services.

A month before her death, Gabriella presented with a recurrence of motor instability, dysphagia, and impaired speech; MRI findings showed disease progression. At that time, the oncologist and the PPC provider conducted a talk on terminality with both parents.

Several multidisciplinary interviews were held with the oncology team, the PPC service, and the parents throughout the course of the disease. Continuity of care was ensured by the early involvement of the pediatric hospice as a facilitator of the post-surgical rehabilitation process. An early care plan of non-invasive emergency treatment had been agreed with the family 6 months before her death.

At home, Gabriella began to have significant respiratory symptoms. Thus, the parents decided, in agreement with clinicians, to admit Gabriella for symptom management. Gabriella died in hospice 2 weeks later, with the whole family close by. See **Table 3** for a summary of the main points of this case.

**Table 3:** Summary of case highlights.

Reason for referral: fatal prognosis with reporting at onset
The PPC team fully introduced themselves
Early referral
Clinical services maintained shared care direction
+ PPC service presentation at a time of postoperative recovery; the constant presence of oncologists at crucial moments of communication - The presence of many extended family members who needed exclusive and dedicated communications

## Case 4—Lisa (early referral and discontinuity of care)

Lisa was a 14-year-old girl with metastatic alveolar rhabdomyosarcoma of the left hand. She was 10 years old at the onset of the disease, lasting a total of 4 years. After diagnosis, Lisa had undergone CT and RT with an initial response. However, over the years, Lisa presented with disease persistence and new subcutaneous nodules for which she received targeted RT. Therefore, the oncologists referred the patient to the PPC service 13 months before her death.

During oncology admissions, the PPC service met Lisa and her mother and was introduced to her as the pain therapy team. The PPC team spoke with her mother and father (by phone from his workplace) to present the service. They immediately expressed their great religious faith and how the family often discussed the meaning of life and death. It emerged that Lisa had asked her parents many questions about her prognosis.

Shared non-invasive emergency care planning was introduced in agreement with family members after the incurability was declared. For the first 8 months after admission, Lisa mostly needed antalgic support for pain and itch management in the disease areas. The PPC team carried out clinical updates with Lisa and her family, mainly during re-evaluations in pediatric onco-hematology.

After this period, Lisa had a progressive worsening in the number and size of the subcutaneous nodules; she was also unable to walk following an injury (with subsequent localization of disease in the femur, pelvis, and spine). During a routine visit, she asked the oncologist and PPC physician about the prognosis and the stage of her disease. Lisa revealed that she searched for information online daily (e.g., by Googling disease characteristics and following young people with similar diseases on TikTok). The girl explained that she wanted to play an active role and learn about all aspects of her disease. The parents separately asked the specialists to remain positive and not to reveal that the disease was incurable. However, over the following 5 months, the communications were always directed to Lisa as the main character instead of just her parents.

The PPC psychologist offered psychological support to all family members, but only Lisa benefited. The family was close-knit but socially isolated. Thus, Lisa began seeing the PPC workers as pleasant visitors. Home care services (e.g., physiotherapy and routine nursing supervision of bandages) were provided at home since Lisa could no longer move from her bed.

Two more hospice admissions were made in the 5 months to facilitate Lisa’s palliative RT sessions and modulation of antalgic therapy. During the last 3 months, parents frequently requested meetings with the oncology team who had been following their daughter for 4 years. The main interlocutors became the palliative clinicians, which complicated the scheduling of a meeting with the oncologists, as requested by the family. The difficulty of seeing the specialists generated a sense of abandonment.

As managing symptoms at home became too challenging for the family, they decided to accept Lisa’s wish to return to hospice. Lisa was at ease with all clinicians but alternated moments of strong discomfort, anger, sadness, and existential angst. She once asked, “no longer receive bad news” and agreed to include mood-stabilizing psychotropic drugs in her therapy. Conversations with the psychologist continued with an intense exploration of the meaning behind the negative experiences and the search for coping strategies. Many conversations centered on religious values and existential meaning. Lisa claimed that she had suffered great injustice but deserved a place in heaven. A great religiousness guided the whole family. Lisa passed away in hospice, as she had asked and desired, with control of her pain and anxiety symptoms. See **Table 4** for a summary of the main points of this case.

**Table 4:** Summary of case highlights.

Reason for referral: type of disease at diagnosis and resistance to current therapy
The PPC team fully introduced themselves to the parents and as pain service to the patient
Early referral
Family members’ feelings of being discharged by the oncologist once the RT was completed
+ Positive therapeutic relationship due to the presence of the PPC team at oncology follow-up visits - Difficulties in oncologists’ involvement in communication with family during EoL hospitalization in pediatric hospice

This summary table (**Table 5**) shows the variability of case management due to their complexity and level of participation in the PPC service. The four cases are very diverse and the table reflects this heterogeneity. The disease histories range from 10 to 48 months, with prolonged treatment periods (between 4 and 42 months). In all cases, palliative care was involved initially for pain treatment. The main reasons for the PPC involvement were different but mostly related to EoL management. The level of participation of the PPC team during conversations also varied. In fact, during aggravation and incurability communications, the PPC team was not always present but was involved whenever there was a communication of terminality. The number of meetings, multidisciplinary interviews with the family, and visits by the PPC team during oncology admissions represented simple and objective indices of integration between services.

**Table 5:** Summary of clinical data and PPC involvement.

		Ida (late referral and discontinuity of care)	Antony (late referral and integration of care)	Gabriella (early referral and integration of care)	Lisa (early referral and discontinuity of care)
Age at diagnosis; years, months		13 yr	2 yr 7mo	5 yr 7 mo	10 yr
Duration of the disease, months		12 mo	14 mo	10 mo	48 mo
Surgery		Yes	Yes	Yes	Yes
Duration of curative treatment (CT/ RT), months		9 mo	12 mo	4 mo	42 mo
Palliative CT/RT		No	Yes	Yes	Yes
Pain management by PPC		Yes	Yes	Yes	Yes
Taking charge of PPC service, months		0 mo	2 mo	8 mo	13 mo
Reason for PPC referral		EoL management	EoL management	Complex symptoms & Incurability	Incurability
Age at death; years, months		14 yr	3 yr 10 mo	6 yr 6 mo	14 yr
Inclusion of PPC’s team into the conversation about	Aggravation	No	No	Yes	No
	Incurability	No	Yes	Yes	Yes
	Terminality	Yes	Yes	Yes	Yes
Patient involved in the communication of terminality		No	No	No	Yes
Multidisciplinary interviews with the family		1	3	5	3
Visits by the PPC team during oncology admissions		0	1	10	17
Psychological interview from PPC’s service		5	1	26	34
PPC professionals’ home visits		0	2	5	5
Follow-up phone calls from the PPC service		0	3	7	14
Deceased in pediatric hospice		Yes	No	Yes	Yes

CT, chemotherapy; EoL, end-of-life; PPC, pediatric palliative care; RT, radiotherapy.

We classified the PPC team interventions into specialized psychological interviews, home visits by PPC professionals, and follow-up phone calls. These data show the involvement of palliative care professionals from different walks of life and at different levels (from practical home support to psychological interviews).

## Discussion

Is it early enough? That alone is not enough. The clinical cases analyzed show that the synergy between oncology and PPC remains crucial.

The referral of Ida and Antony to PPC occurred later, as evidenced by the partial involvement of PPC in their care plan. This delayed introduction posed challenges in pain management for Ida and resulted in a costly cycle of palliative CT for Antony and his family. Nevertheless, an effective integration of services was achieved, which made it possible to fulfill the parents’ wish for Antony to spend his final moments at home. Conversely, the cases of Gabriella and Lisa exemplify an early referral to PPC. The patients were referred to palliative care during clinical stability, fostering trust between the families and the PPC service. While Gabriella’s case involved seamless synergy between services, the perception of Lisa’s parents was that direct communication with oncologists was lacking, and the PPC team primarily mediated the interactions. This underscores the necessity for more than just early referral to ensure effective integration between oncology and palliative care. Timely intervention is crucial, but showing synergy and ongoing open discussion is essential to employ the best strategies to ensure the highest possible quality of life.

Based on a literature review and real-world case analysis, the following discussion outlines key recommendations for early PPC involvement in pediatric oncology.

We propose a summary of the most critical component and a practical application based on the “Five Ws” (plus one) to promote care integration.

### Who? The team as a vessel

Among the numerous models of PPC integration in oncology described, the best one is the interdisciplinary approach in which different specialists with unique experiences and expertise collaborate as a team to improve overall patient care (9).

The vessel metaphor may help describe to the family the need for a multidisciplinary team to achieve a common goal (10). In addition, as a fluctuating system, palliative care is coordinated between hospital, territory, and home, facilitating a continuum of care; if structured early, this can help to constantly monitor the patient’s needs and detect changes from the baseline (11).

Based on clinical cases, the patient and their family benefit from meeting with the extended team of oncologists and palliative care specialists. Much coordination and collaboration work is submerged; it must appear more visible and tangible.

### What? Referral protocols and guidelines

The ability to maintain a dialogue also comes through speaking the same language; therefore, using shared tools and protocols is crucial. In pediatric oncology, the Pediatric Palliative Screening scale has been validated to be practical without specifying individual prognosis (12). Another tool attracting interest in pediatric emergency departments is the Pediatric Early Warning System. The innovation of this score is that it takes into account the opinion of the parent and the nurse, who are often the figures most in contact with the child (13). The early detection of worsening situations can open up reflection on communication and decision-making, including addressing emergencies in pediatric oncology with possible acute PPC involvement (14). Lastly, the Accertamento dei bisogni Clinico-Assistenziali Complessi in PEDiatria (ACCAPED) scale is an easy-to-apply score dedicated to the assessment of clinical complexity and eligibility for PPC service (15).

The cases illustrate how a consensus among professionals on PPC eligibility criteria prevents family reticence bias and minimizes clinicians’ subjective interpretations.

### When? PPC is a holistic health approach to living and not dying

WHO has strongly recommended that palliative care for children with cancer should begin at diagnosis, regardless of prognosis (5, 16, 17), emphasizing the role of PPC in improving the overall quality of life of children and families living in life-threatening situations. Palliative care does not end with the death of the child but can be a temporary assistance until the resolution of the disease (18). Referral to PPC should be based on needs rather than life expectancy (19). Furthermore, PPC should not be seen as an alternative to cancer treatment but can be provided alongside specific treatments. Continuing direct therapies even at the EoL has a beneficial effect (“doing something”), as reported by parents and young adults (20).

We shall consider the introduction of PPC during disease in case of unresponsive tumors, relapse or progressive diseases, tumor-related severe comorbidities or secondary to treatment toxicity, and prolonged hospitalization in the pediatric intensive care unit (21, 22).

As shown in the clinical cases, the difficulty in establishing a therapeutic relationship between the PPC team and the family depends on the stage of the disease at which it is introduced. In the cases where clinical stability was more significant, it was easier to introduce the PPC service.

### Where? The flexibility of the care setting

Patient care is not confined to the hospital but is integrated into all settings surrounding the patient. Therefore, community and hospital services must constantly integrate to ensure holistic care for the child and their family. One of the strengths of the PPC concept is that it can be delivered anywhere through the support of the community hospital and local home care systems (23, 24). Cultural and social variables are also relevant, especially when patients are foreigners or are political refugees. This means that they may wish to return to their places of origin and that their social needs vary widely. The analysis of the psycho-social needs of the family is fundamental.

In all the cases reported, the primary factor that renders PPC so tangibly valuable is the assistance provided outside the hospital, including all patient’s life settings.

### Why? Rationale and ethics of introducing PPC

When patients need palliative care, appropriate communication should be planned with the family and both teams on the meaning and role of PPC (25). This can help the family deal with the situation from the beginning and externalize needs related to the physical domain. Even in the early stages of the disease trajectory in oncology, the complexity and burden of symptoms that a patient may experience may justify the integration of PPC as supportive care over time (26). In addition, appropriate treatment of symptoms by palliative specialists may reduce the risk of intensive and unnecessary care (27).

One of the main challenges is the meaning of palliative care in common parlance. Parents are often frightened of our involvement in palliative care; therefore, clinicians have the responsibility to help families understand the role of PPC and prevent the perception that one excludes the other (28). A transparent definition of roles between oncologists and palliative care providers and a clear explanation to the family about how and who is activated when needed help families and physicians maintain a strong collaboration between the teams. There has been some discussion about the possibility of reformulating the term “palliative care”, but this concept has yet to be more debated (29, 30).

The PPC service is often presented as a pain management service, highlighting the difficulty of using a term frequently linked to the EoL. Several research studies have shown that using expressions other than palliative care could help consider this service a health promotion service (30, 31).

In all the cases illustrated, PPC was effectively presented as supporting treatment and pain management. In clinical practice, PPC is introduced as a service that promotes quality of life, which should be enhanced at any disease stage.

### Extra: to whom? Keeping the focus on communication as a time for healing

These cases demonstrate how team communication should constantly share the care pathway and support the family. Indeed, the oncology team is usually the essential first point of reference. Clinicians are professionals whom the family trusts and from whom they will always seek advice (32, 33). The first year after diagnosis is challenging because of the changes the family and the patient must manage; in this phase, the relationship with the care team is crucial (34). This solid therapeutic relationship facilitates communication, particularly the delivery of bad news. However, oncology follow-ups and contact with the family may decrease once the PPC service is involved. The adult medicine literature shows how this change produces a perception of abandonment, as confirmed by some research on adult medicine (3, 5). The family may also suffer from the loss of the hospital places and staff, who have become almost a second family (35). The sharing of the patient and their family caretaking by oncologists with PPC specialists is an empathetic moment that requires developed communication skills (36).

Pediatric oncological disease leads the family to oscillate between a pole of increased hope and one of contemplation of death. Knowing the family’s hopes and fears provides a better understanding of the challenges the family is facing and fosters open and honest clinical communication (37, 38).

It became clear that more efforts need to be made to ensure that patients know that being informed about their health is compatible with their age. The family frequently asks not to communicate incurability to avoid taking away hope or generating despair. Communication in pediatrics concerns the balance in the clinical–parent–patient triad (39–41).

In the cases analyzed, psychological support is available only sometimes or at certain times in the history of the disease. It is not always provided on an ongoing basis, and not all parents accept support for themselves, let alone for their children. Psychological support is a standard of care in pediatric oncology (42, 43); it reduces stress and helps develop coping mechanisms (44). Psychological support should also be provided to siblings of patients with cancer.

In future developments, comparing realities from different countries and evaluating clinical cases with more objective data (e.g., questionnaires, psychological tests, pain, and symptom rating scales) would be stimulating.

## Conclusion

Ensuring continuity of care can be challenging despite existing gold standards and best practices. More than an early referral is required. Collaborative teamwork must be implemented consistently in clinical practice, requiring coordination and open communication. Cultural barriers often lead families to hesitate to seek or decline palliative care, underscoring the need for open communication and regular case reviews. This article emphasizes the importance of standardized dialogue between teams and pediatric patients and their families.

## Data availability statement

The original contributions presented in the study are included in the article/supplementary material. Further inquiries can be directed to the corresponding author.

## Ethics statement

Written informed consent was obtained from the individual(s) for the publication of any potentially identifiable images or data included in this article.

## Author contributions

AS: Conceptualization, Data curation, Supervision, Writing – original draft, Writing – review & editing. IA: Conceptualization, Data curation, Methodology, Writing – original draft. MA: Supervision, Writing – review & editing. AZ: Data curation, Methodology, Supervision, Writing – review & editing. FB: Conceptualization, Supervision, Validation, Writing – review & editing.
